# An electrospun poly(ε-caprolactone) nanocomposite fibrous mat with a high content of hydroxyapatite to promote cell infiltration

**DOI:** 10.1039/c8ra02059k

**Published:** 2018-07-16

**Authors:** Haoxuan Li, Chen Huang, Xiangyu Jin, Qinfei Ke

**Affiliations:** Key Laboratory of Textile Science & Technology, College of Textiles, Donghua University No. 2999 North Renmin Road, Songjiang Shanghai 201620 P. R. China kqf@dhu.edu.cn

## Abstract

Electrospun polymer/inorganic biomimetic nanocomposite scaffolds have emerged for use in a new strategy for bone regeneration. In this study, a poly(ε-caprolactone) (PCL)/hydroxyapatite (HAp) nanocomposite mat with a HAp content as high as 60% was prepared *via* one-step electrospinning using trifluoroethanol as the solvent, and it has superior dispersibility and spinnability. The structure and physicochemical properties of the scaffolds were studied using scanning electron microscopy and spectroscopic techniques. X-ray diffraction and Fourier transformed infrared spectroscopy confirmed the presence of HAp in the composite PCL fibers. The results of cell culturing suggested that the incorporation of HAp with PCL could regulate the cytoskeleton and the differentiation of cells. More interestingly, the high content of HAp was also found to be conducive to the infiltration of MC-3T3 cells into the mat. The results indicated the potential of PCL/HAp scaffolds as a promising substitute for bone regeneration.

## Abstract 2

PCL nanofibers with 60% HAp content were fabricated, and the presence of HAp regulated cell morphology to enhance cell infiltration.

## Graphical abstract

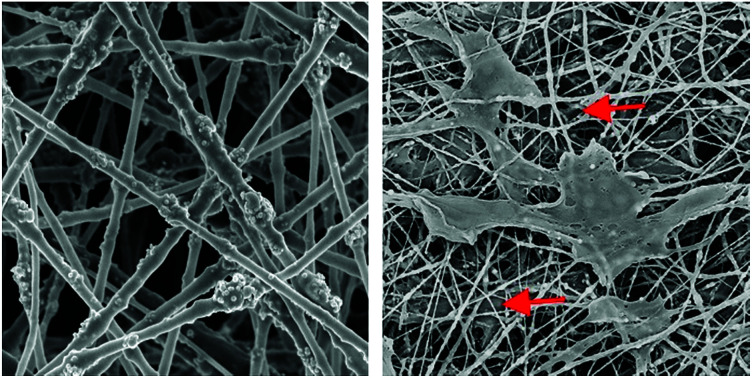

## Introduction

Large bone defects require reconstructive surgeries as the recovery process is beyond the capability of a human to self-repair.^1,2^ An autograft is still the golden standard for bone defect treatment,^3^ although the method shows limitations, such as pain, hemorrhage and infection at the donor site. To overcome these problems, research into inducing natural regeneration of bone *via* a tissue engineering strategy has emerged in recent years.^4–7^ The demands for an ideal bone scaffold should include superior biocompatibility and suitable mechanical properties, and it should mimic the structure and composition of bone.^8^

Electrospinning is a simple and versatile way to fabricate nano- or micro-scale fibers, and it has gained popularity in tissue engineering in the last decade. The scaffolds produced *via* electrospinning have a unique structure with interconnected pores and a high surface to volume ratio, and exhibit a high similarity to the natural extracellular matrix (ECM).^9–11^ In particular, the feasibility of incorporating functional inorganic nanoparticles into nanofibers to meet special requirements, such as bone regeneration, has made electrospinning very attractive. The natural bone matrix is composed of 65% mineral and 35% protein. The mineral phase is predominantly hydroxyapatite (HAp).^12^ Therefore, many researchers are devoted to preparing biodegradable polymer-based scaffolds containing HAp to mimic the structure and function of the ECM of bone as closely as possible.^13–16^ However, due to the agglomeration of HAp particles, the fabrication of uniform nanofibers with a higher HAp concentration (over 40%) still remains a big challenge.^17^ Xia’s group reported a method to coat calcium phosphate on a mat of electrospun nanofibers by adding 10 times concentrated simulated body fluid (10SBF).^18–20^ However, the nanofibers became microfibers after the mineralization, since there was a relatively thick layer of calcium phosphate on the nanofibers. When Li^21^ utilized γ-glycioxypropyltrimethoxysilane (known as A-187) to modify HAp nanoparticles to improve their dispersion in the PCL matrix, the content of HAp in PCL could reach 30%. Therefore, it is necessary to explore a simple and effective approach to increase the content of HAp in electrospun polymer nanofibers. Besides, another issue with electrospun scaffolds for bone tissue engineering is cell infiltration.^22^ During cell culturing, the nanofibrous scaffolds showed a superior attachment and proliferation of cells but a lack of penetration in depth. This is because the diameter of the nanofibers and the pore sizes of the nanofibrous scaffolds were too small to allow cell penetration into the scaffolds.^22^ To date, many researchers have explored ways to increase the pore size of the electrospun fibrous mat to promote cell infiltration.^23,24^ However, few studies have investigated how to reduce the size of the cells by regulating the material’s morphology to improve the infiltration of cells.

**Fig. 1:**
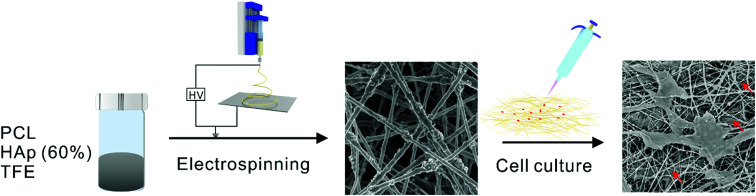
Schematic diagram of the effect of a high concentration of HAp in PCL nanofibers on the infiltration of MC-3T3 cells.

Herein, we report the effect of a high concentration of HAp in PCL nanofibers on the infiltration of MC-3T3 cells (Fig. 1). Polycaprolactone (PCL) is a synthetic biomaterial, which is widely applied in tissue engineering scaffolds because it has superior biocompatibility and a good spinnability.^25^ PCL composite nanofibers with high concentrations of HAp, as high as 60%, were successfully prepared *via* electrospinning, which is close to the amount in the mineral component of natural bone.^26^ The structure and physicochemical properties of the materials were evaluated, and the effect of different concentrations of HAp on the cellular proliferation and infiltration was also investigated.

## Experimental

### Materials

Polycaprolactone (PCL, average *M*_n_ = 80 000), 2,2,2-trifluoroethanol (TFE, 98%), hydroxyapatite (HAp, nanoparticles, <200 nm particle size (BET), ≥97%), and all chemicals used for *in vitro* experiments were purchased from Sigma-Aldrich (St. Louis, USA). MC3T3-E1 cells were purchased from the Institute of Biochemistry and Cell Biology (Chinese Academy of Sciences, China).

### Preparation of electrospun fibrous mats

PCL/HAp electrospun solutions were prepared by adding 1.2 g PCL into 10 mL TFE to get a 12% (w/v) PCL/TFE solution. Then 0.5 g and 1.8 g of HAp nanoparticles were added into two bottles of 12% (w/v) PCL/TFE solution, separately. All the solutions were magnetically stirred at room temperature for 24 h for better homogenization to generate hybrid suspensions with different amounts of HAp (30 and 60%). The solution was loaded into a 5 mL plastic syringe with a 21-gauge needle attached and was dispensed using a syringe pump. The injection rate was maintained at 1 mL h^−1^. The distance between the tip of the needle and the collector was about 15 cm and a voltage of 15 kV was applied.^27,28^

The content of HAp in the PCL/HAp nanofibrous mats was calculated using the following formula:
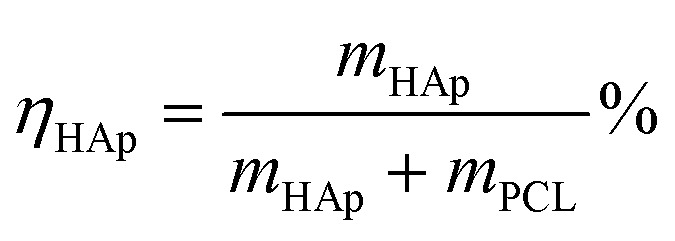
where *η*_HAp_ is the content of HAp in the PCL/HAp nanofibrous mat, and *m*_HAp_ and *m*_PCL_ are the mass of HAp and PCL, respectively. Therefore, as 0.5 and 1.8 g of HAp were added into 10 mL TFE solution with 1.2 g PCL, separately, the content of HAp in the HAp/PCL nanofibrous mats is about 30% and 60%.

### Characterization

The morphology of the electrospun PCL/HAp nanofibrous mats was viewed using a field emission scanning electron microscope (FE-SEM) (JSM7500F, JEOL, Japan) at an accelerating voltage of 5 kV. The pore size and contact angle of the mats were measured using a Capillary Flow Porometer (CFP-1100-A) (PMI, USA) and a Surface Tension Meter (XG-CAMA1, China). The mechanical properties of the pristine and welded nanofiber mats were measured on an ELF 3200 uniaxial testing system (Bose, Germany). Samples from the two groups were cut into testing strips of 0.25 cm × 1 cm, with a thickness of 10 μm. The samples were tested under uniaxial tension under quasi-static conditions, with a constant tensile speed of 1 mm s^−1^ along the direction of motion. X-ray diffraction (XRD) patterns and FT-IR spectra in ATR mode were obtained using an X’Pert PRO Alpha-1 diffractometer (PANalytical, The Netherlands) and a Nicolet 6700 infrared spectrophotometer (Thermo Fisher, USA).

### MC3T3-E1 cell cultures and seeding

For culturing, MC3T3-E1 cells were grown in α-Minimum Essential Medium (α-MEM, Gibico) supplemented with 10% fetal bovine serum (FBS, Gibico) and 1% (v/v) antibiotics (containing penicillin and streptomycin, Gibico). Prior to cell seeding, the scaffold was sterilized in 75% ethanol for 1 h, washed with phosphate-buffered saline (PBS, pH = 7.4, Thermo Fisher) three times and immersed in α-MEM overnight. For cell seeding, 100 μL of the medium containing 20000 MC-3T3 cells and 400 μL culture medium were pipetted onto the center of the sample. The cell seeded samples were placed in an incubator and kept at 37 °C and 5% CO_2_ for 1 hour. Subsequently, 4.5 mL culture medium was added to each well. The culture medium was changed every other day.

### Cell proliferation and differentiation

To evaluate the proliferation, the cells were cultured in 24-well plates for 14 days. At each time point (1, 3, 7 and 14 days), 3 samples were tested for each composite mat, and the culture medium was removed and the samples were washed with PBS solution three times. After this, 360 μL FBS-free α-MEM and 40 μL 3-(4,5-dimethyl-2-thiazolyl)-2,5-diphenyl-2-*H*-tetrazolium bromide (MTT) solution in PBS (5 mg mL^−1^) were added to each well and incubated for 4 h. The culture medium was then withdrawn and 500 μL dimethyl-sulfoxide (DMSO) was added. Subsequently, the plate was incubated for 30 min in a shaker. When the formazan crystal had dissolved completely, 100 μL of the solution was pipetted into a 96-well plate and tested at 492 nm using a microtiter plate reader (Multiskan MK3, Thermo, USA).

Alkaline phosphatase (ALP) staining was performed using a Vector Red Alkaline Phosphatase Substrate Kit (SK-5100, Vector Laboratories, Burlingame, CA) according to the manufacturer’s instructions. ALP activities were quantified on the basis of the mean pixel intensity using Image J. Three samples from each group were analyzed at each time point.

### Cell viability assay

The cell morphology was observed using a scanning electron microscope (SEM, JEOL JSM-5600, Japan) at an accelerating voltage of 15 kV. After 1, 3, 7 and 14 days of culturing, the medium was removed from the wells and the samples were washed with PBS solution three times. Then, the samples were fixed with 4% paraformaldehyde (PFA) for 30 min at 4 °C. The samples were dehydrated with gradient ethanol (30, 50, 70, 80, 90, 95 and 100% v/v) for 10 min. Finally, the samples were freeze-dried overnight and coated with gold in an automatic sputter coater, and were then observed using SEM.

The morphology and cytoskeleton of the adherent cells were observed using confocal laser microscopy imaging. After 3 days of culturing, the samples were rinsed with PBS twice and then fixed with 4% paraformaldehyde for 30 min at 4 °C. Then, the samples were incubated in 1% Triton X-100 (Sigma, USA) for 10 min to permeate the cell membrane. After being washed with PBS 3 times, the cytoskeletons and nuclei of the cells were stained with rhodamine-conjugated phalloidole (25 μg mL^−1^) and 4′,6′-diamidino-2-phenylindo hydrochlorides (10 μg mL^−1^) (Invitrogen, USA) for 30 min. The samples were observed using laser scanning confocal microscopy (LSCM) (Carl Zeiss, Germany).

### Statistics

The results are presented in the form of mean ± standard deviation, with “*n*” indicating the number of samples per group. The comparison between the groups was analyzed using Student’s *t*-test in SigmaPlot. The statistical significance was set to *p* < 0.05.

## Results and discussion

**Fig. 2:**
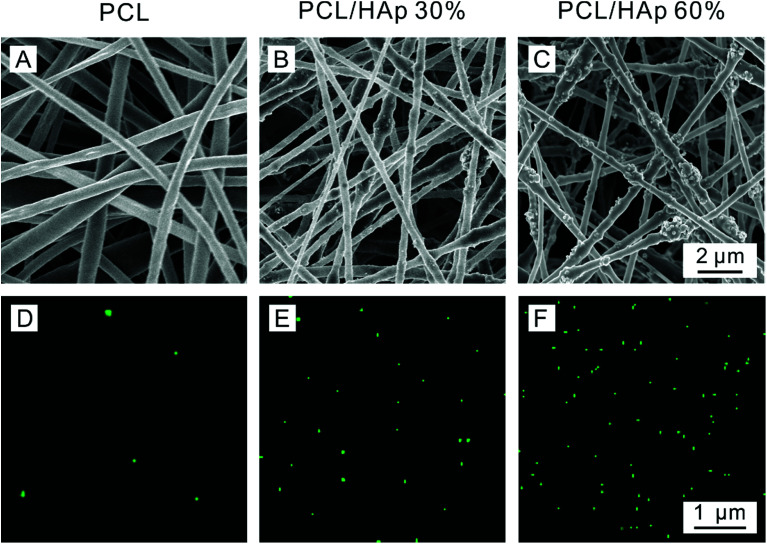
SEM images and distributions of calcium of the PCL/HAp random nanofibrous mats: (A and D) pure PCL, (B and E) PCL/HAp 30%, and (C and F) PCL/HAp 60%.

Fig. 2 shows scanning electron microscopy (SEM) and Energy Dispersive X-ray (EDX) mapping images that were taken of the PCL/HAp nanofibrous mats with different amounts of HAp. For comparison, pure PCL nanofibers with a smooth surface were produced and the diameter of the fibers was approximately 393 ± 100 nm (Fig. 2A). As shown in Fig. 2B, the HAp/PCL nanofibers with 30% HAp were successfully fabricated with uniform and continuous fiber morphology. A rough surface on the nanofibers was observed, which is probably because the HAp nanoparticles were encapsulated in the PCL or were exposed on the surface of the nanofibers. When the content of HAp was increased to 60%, continuous and homogeneous nanofibers were also fabricated (Fig. 2C), owing to the excellent electrospinnability of the PCL/TFE solution system. Excluding the exposed HAp, the diameter of the HAp/PCL fibers was about 317 ± 40 nm for 30% HAp and 332 ± 44 nm for 60% HAp, and these were both smaller than the diameter of the pure PCL nanofibers, indicating that the incorporation of HAp had essentially no significant influence on the diameter of the PCL nanofibers. Varying the concentration of HAp affects the viscosity of the solution, resulting in a decrease in fiber diameter in the presence of HAp. More detailed information can be found in Table 1. It should be pointed out that the incorporation of HAp into PCL nanofibers does not significantly influence the pore size and contact angle of the electrospun PCL/HAp nanofibrous mats. Distributions of calcium across the pristine PCL (Fig. 2D), PCL/HAp with 30% HAp (Fig. 2E) and PCL/HAp with 60% HAp (Fig. 2F) nanofibrous mats were revealed using energy-dispersive X-ray spectroscopy (EDX) mapping. As anticipated, the distribution of calcium increased from pure PCL to the PCL/HAp nanofibrous mats. This result was consistent with the increase of HAp in PCL nanofibers, suggesting that the HAp nanoparticles were successfully loaded into the PCL nanofibers *via* electrospinning. It should be pointed out that, in contrast to mineral deposition, the incorporation of the HAp nanoparticles did not significantly influence the diameter of the electrospun nanofiber, and the nanoscale of the fibers was maintained. Taken together, these results suggest that our approach offers a simple and versatile method for fabricating an electrospun nanofibrous mat with a high content of HAp.

**Table 1:** Detailed information for the PCL/HAp nanofibrous mats with different amounts of HAp

Sample	Areal density (g m^−2^)	Average pore size (μm)	Diameter (nm)	Contact angle (°)
PCL	12.34	1.944	393 ± 100	135.4
PCL/HAp 30%	17.86	2.187	317 ± 40	134.1
PCL/HAp 60%	28.43	2.297	332 ± 44	131.8

**Fig. 3:**
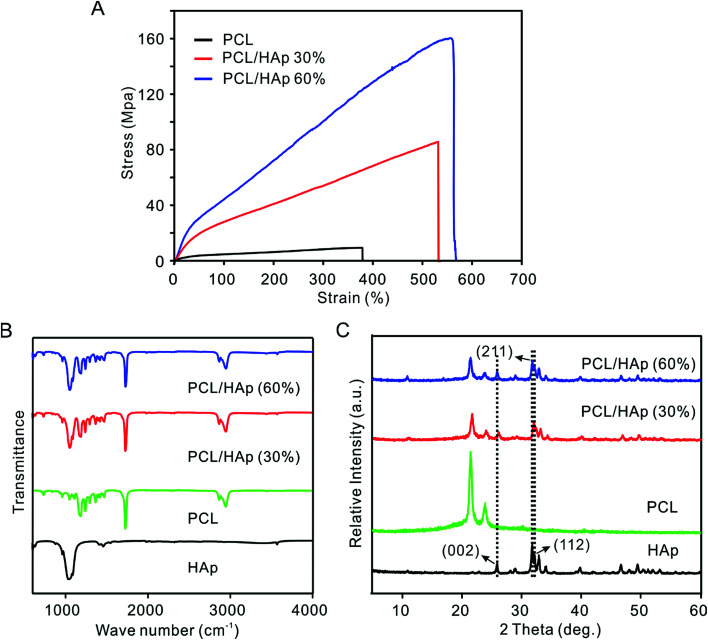
(A) Stress–strain curve of the nonwoven mats of the pristine and PCL/HAp random fibers. (B) FTIR spectra and (C) XRD patterns of the HAp nanoparticles, PCL and PCL/HAp fibrous mats.

Fig. 3A shows the stress–strain curves of PCL and the PCL/HAp nanofibrous mats. The PCL/HAp nanofibrous mat containing 60% HAp exhibited the highest tensile strength (158.1 ± 12.6 MPa) compared to a strength of 12.3 ± 0.89 MPa for the pristine PCL and 85.17 ± 2.61 MPA for PCL/HAp nanofibrous mats with 30% HAp, suggesting a greatly enhanced tensile strength after combination with HAp. The same trend was observed for elongation as well: the elongation increased from 380% to 530% and 564% when the HAp was increased from 0 to 30 and 60%, respectively, indicating that the introduction of HAp enhanced the ability to absorb energy before the failure of the nanofibrous mats. Notably, the occurrence of HAp could mimic a favorable mechanical environment for bone regeneration. The presence of HAp nanoparticles in PCL nanofibers was detected using FTIR and XRD (Fig. 3B and C). The pure PCL spectrum showed three main bands: the stretching vibrations of the carboxyl (C=O) at 1726 cm^−1^, the stretching vibrations of the ether groups (C–O–C) at 1180 cm^−1^ and the symmetric stretching of C–H at 2867 cm^−1^. In addition to the PCL characteristic bands, the PCL/HAp spectra also showed the characteristic bands of nanophase HAp. The P–O vibrational (603 cm^−1^) band, OH– vibrational (631 cm^−1^, 3572 cm^−2^) bands and PO_4_^3−^ (963 cm^−1^, 1040 cm^−1^) bands, are attributed to the combination of PCL and HAp. The spectrum of HAp showed two absorption bands at 1413 cm^−1^ and 1459 cm^−1^, which are a sign of CO_3_^2−^ entering the apatite structure.^29^ When compared to the PCL nanofibers, the XRD spectra of the PCL/HAp composite nanofibers showed three new peaks at 26.1 (002), 31.7 (211) and 32.4 (112), which could be attributed to the characteristic diffraction angles of crystalline HAp. The same peaks were also observed in the XRD diffraction pattern of the HAp nanoparticles.

**Fig. 4:**
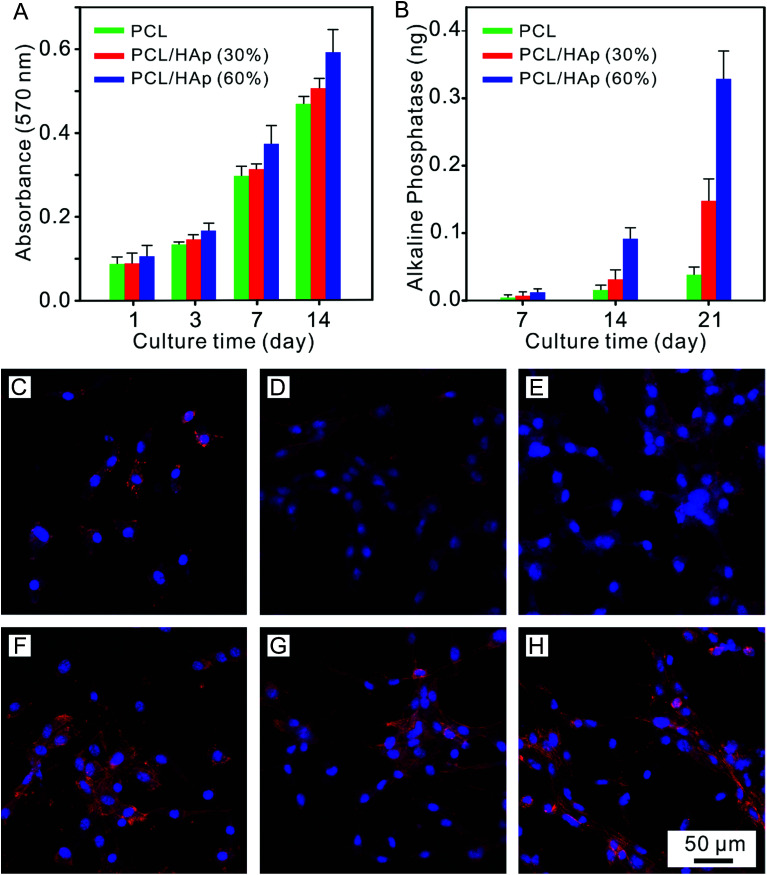
(A) MC 3T3 cell proliferation on the PCL/HAp fibrous mat with a graded mineral for 1, 3, 7 and 14 days. (B) ALP activity of the MC 3T3 cells seeded on the PCL/HAp fibrous mat with a graded mineral for 7, 14 and 21 days. Confocal images of the MC-3T3 cells cultured on the (C and F) PCL, (D and G) PCL/HAp (30%), and (E and H) PCL/HAp (60%) fibrous mat after 1 day and 3 days, respectively.

In order to evaluate the effects of the nanofibrous mat with different mineral amounts on the cell response, pre-osteoblast MC-3T3 cells were seeded onto these mats, which were cultured using either proliferation medium or osteogenic medium for 1, 7, 14 and 21 days. Firstly, we analyzed the proliferation rate of the MC-3T3 cells on the HAp/PCL nanofibrous mat with a HAp content of 0%, 30% and 60% after 1, 3, 7 and 14 days of culturing. As shown in Fig. 4A, a significant difference between pure PCL and PCL/HAp 60% was found at day 1. This can be explained by the idea that the larger surface area provided by the greater surface roughness resulted in more cell binding cues from the mats. It can be noticed that a gradient in cell density, positively correlated with the mineral content, began to appear at day 7 and became more prominent with increasing culture time. The mats with the highest HAp content showed the highest level of cell density, while the mats with lower HAp content had fewer cell numbers. It can be noticed that the proliferation rate on the PCL/HAp 60% nanofibrous mat was significantly higher than that on pure PCL and PCL/HAp 30% after 14 days of culturing. This phenomenon demonstrated that, compared to pure PCL, the incorporation of HAp into the PCL nanofibers showed a promotion in the proliferation of the cells. These results showed that the MC-3T3 cells preferentially proliferate on the regions with higher HAp content. Then, the alkaline phosphatase (ALP) expression was investigated to understand the influence of HAp on the MC-3T3 differentiation. The intensities of the ALP expression on different mats are shown in Fig. 4B, and it can be seen that there was a positive correlation between the ALP expression and the HAp content and culture time. Regardless of culture time, the HAp content had a significant effect on ALP expression. These results indicated that incorporation of HAp into PCL nanofibers can enhance the differentiation of MC-3T3 cells.

**Fig. 5:**
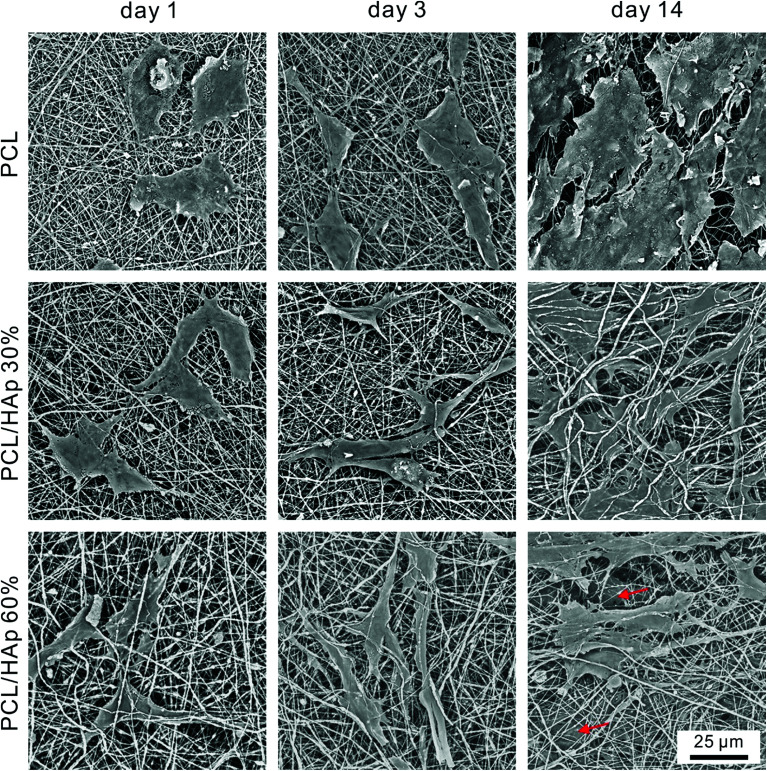
SEM images of the MC-3T3 cells cultured on the PCL, PCL/HAp (30%), and PCL/HAp (60%) fibrous mats after 1 day, 3 days and 14 days, separately. The red arrows indicate the infiltrated cells.

To further study the morphology and cytoskeleton of the adherent cells on the graded mats, we then examined the cytoskeletal organization of the cells attached on the mats with different amounts of HAp after 1 and 3 days of culturing. Fig. 4C shows the confocal image of the MC-3T3 cells on the pure PCL mat at 1 day after seeding, and the image indicates that the cells maintained a spherical morphology with no extending filopodia. Conversely, more stretched MC-3T3 cells at the PCL/HAp mat with HAp 30% and 60% were found to spread along the nanofibers (Fig. 4D and E), suggesting that the combining of HAp into the PCL mat dramatically increased the cellular attachment and spreading. At day 3, the cells on the pure PCL mats were less stretched (Fig. 4F) than those on the PCL/HAp mats, which displayed a spindle-shaped elongated morphology and became more prominent, suggesting the general formation of actin fibers (Fig. 4G and H). These results are consistent with the previous report.^30^ The significant difference in the morphology of the MC-3T3 cells may be attributed to the rough surface structure of the nanofibers or the chemical composition owing to the incorporation of HAp. All of these results suggested that the cell response to the nanofibrous mat, such as proliferation, differentiation and morphology, can be regulated by embedding HAp nanoparticles into PCL nanofibers. We then observed the morphology of long term cultured cells on the graded mat using SEM. As shown in Fig. 5, the MC-3T3 cells showed different morphologies on the PCL nanofibrous mats with different amounts of HAp. After 1 day of culturing, a gradient in the cell elongation positively correlated with the HAp content. The morphology of the cells on the pure PCL mat exhibited isotropic extension, while the cells on the PCL/HAp mat exhibited anisotropic elongation. These phenomena became more prominent with increasing culture time. After 14 days of culturing, on the pure PCL mat, cells covered the entire surface of the mat, leaving less space for further growth. Interestingly, two cell layers on the surface and interior were observed on the PCL/HAp nanofibrous mats with 60% HAp (as marked by the red arrow in Fig. 5), and this observation corresponds with our proliferation result. It could thus be concluded that the presence of HAp could enhance the elongation of cells, resulting in the size of the elongated cells being smaller than the pore size of the mat. This is why the cells could spread into the interior of the nanofibrous mat. To the best of our knowledge, the relationship between cell infiltration and HAp proportion in electrospun fibers has been studied for the first time. In spite of various methods for increasing the pore size of electrospun scaffolds to promote cell infiltration, our results showed that cell penetration could be achieved by simply adding HAp nanoparticles into the composite scaffold. These data prove that the PCL/HAp nanofibrous mats containing 60% HAp provide a better host environment for cellular differentiation and infiltration than the pure PCL nanofibrous mat.

## Conclusions

In conclusion, PCL nanofibrous mats with different amounts of HAp were fabricated *via* one-step electrospinning. The HAp nanoparticles were homogeneously distributed inside PCL nanofibers, and the proportion of HAp in a PCL nanofiber could reach a high record of 60%. More importantly, there were no significant differences in the diameter of the PCL fibers after the incorporation of HAp. Cell viability and infiltration were studied *in vitro* using MC-3T3 pre-osteoblasts cells. The results showed that the PCL/HAp 60% nanofibrous mat was optimal for the attachment, proliferation and spread of MC-3T3 cells. Besides this, the proliferation rate, the expression of ALP and the elongation of cells were found to be positively correlated with the HAp content, resulting in a spatial gradient of cell phenotypes. Infiltration of cells into the mat was observed after 14 days of cell culturing and two cell layers were observed when the proportion of HAp was high (60%), suggesting that the PCL/HAp 60% nanofibrous mat could be a potential candidate for bone tissue engineering.

## Conflicts of interest

The authors declared no potential conflicts of interest with respect to the research, authorship and/or publication of this article.

## Supplementary Material
